# A Laboratory-Based Surveillance Study of Invasive *Neisseria meningitidis*, *Streptococcus pneumoniae*, and *Haemophilus influenzae* Diseases in a Serbian Pediatric Population—Implications for Vaccination

**DOI:** 10.3390/diagnostics11061059

**Published:** 2021-06-09

**Authors:** Snezana Delic, Vera Mijac, Ina Gajic, Dusan Kekic, Lazar Ranin, Boris Jegorovic, Davor Culic, Valentina Cirkovic, Marina Siljic, Maja Stanojevic, Metka Paragi, Milos Markovic, Natasa Opavski

**Affiliations:** 1 Centre for Microbiology, National Reference Laboratory for Meningococcus and Haemophilus, Institute of Public Health, 25101 Sombor, Serbia; mikrobiologija@zzjzsombor.org (S.D.); culicd@yahoo.com (D.C.); 2 Faculty of Medicine, Institute of Microbiology and Immunology, University of Belgrade, 11000 Belgrade, Serbia; ina.gajic@med.bg.ac.rs (I.G.); dusan.kekic@med.bg.ac.rs (D.K.); lazar.ranin@med.bg.ac.rs (L.R.); valentinanikolic85@gmail.com (V.C.); marinasiljic@gmail.com (M.S.); maja.stanojevic@med.bg.ac.rs (M.S.); milos.markovic@med.bg.ac.rs (M.M.); natasaopavski@gmail.com (N.O.); 3 National Reference Laboratory for Streptococci, Faculty of Medicine, Institute of Microbiology and Immunology, University of Belgrade, 11000 Belgrade, Serbia; 4 Clinical Centre of Serbia, University Hospital for Infectious and Tropical Diseases, 11000 Belgrade, Serbia; boris.jegorovic@ymail.com; 5 National Laboratory of Health Environment and Food, Department for Public Health Microbiology, 1000 Ljubljana, Slovenia; metka.paragi@gmail.com

**Keywords:** *Neisseria meningitidis*, *Streptococcus pneumoniae*, *Haemophilus influenzae*, invasive diseases, seroepidemiology, molecular epidemiology, conjugate vaccines

## Abstract

The aim of this study was to present the epidemiology of invasive diseases caused by *Neisseria meningitidis* and *Streptococcus pneumoniae* in the pre-vaccine period, and *Haemophilus influenzae* in the post-vaccine period in a pediatric population from Serbia. Among the meningococci, serogroup B dominated (83%), followed by serogroup C (11.3%). High antigenic diversity was found, with fine type P1.5-1,10-4 being the most frequent. Moderate susceptibility to penicillin was common (55%). Within pneumococci, serotypes 19F, 14, 6B, 6A, 18C, 23F, 3, and 7F prevailed, while 19A was rare (3.6%). The coverages of PCV10 and PCV13 were 68% and 84%, respectively. Major sequence types were ST320, ST15, ST273, ST271, and ST81. Non-susceptibility to penicillin (66.7%), cefotaxime (37%), and macrolides (55%) was predominantly detected in vaccine-related serotypes. Among the 11 invasive *H. influenzae* isolates collected, there were six Hib, three non-type b, and two non-typeable strains (ntHi) that were antibiotic susceptible. These results imply a potential benefit of future Men-B vaccine implementations. For pneumococci, as PCV10 was recently introduced, a significant reduction of morbidity and antibiotic resistance might be expected. The efficiency of Hib vaccination is evident, but a shift towards non-type b and ntHi strains may be anticipated.

## 1. Introduction

*Neisseria meningitidis*, *Streptococcus pneumoniae*, and *Haemophilus influenzae* are important human pathogens causing invasive life-threatening infections that are a major cause of morbidity and mortality in childhood. Variations in the chemical composition of capsular layers of these bacteria lead to immunologically diverse types. Meningococci are grouped within 12 serogroups, with the majority of invasive infections caused by strains belonging to A, B, C, Y, W-135, and X serogroups. In pneumococcus, more than 90 capsular serotypes have been described and serogroups 1, 6, 14, 19, and 23 were among the most common causes of bacteriaemia, sepsis, meningitis, and other invasive pneumococcal diseases (IPD) in children [1]. Regarding *H. influenzae*, significant invasive potential is related primarily to capsular type b (Hib).

Epidemiology of meningococcus, pneumococcus, and *H. influenzae* varies significantly between countries and regions. Serotypes/serogroups differ in invasive potential, propensity towards specific disease, antibiotic resistance profiles, geographic and temporal distribution, between age groups, etc. [1,2]. However, their distribution has been most profoundly affected by the availability of vaccines, especially conjugate vaccines used in wide-scale immunization programs for children that are a principal reservoir of these pathogens in the population [3,4,5]. The conjugate Hib vaccine was first developed in the late 1980s, followed by conjugate pneumococcal and meningococcal vaccines introduced in the late 1990s and early 2000s [3,6,7]. For pneumococci, there are two currently used pneumococcal conjugate vaccines (PCV), 10-valent (PCV10) and 13-valent (PCV13, which replaced the 7-valent vaccine, PCV7) that target the serotypes most frequently encountered in invasive infections, as well as the serotypes exhibiting antimicrobial resistance [7]. Different formulations of meningococcal conjugate vaccines against A, C, Y, and W135 serogroups are available, while a recombinant vaccine for B group has recently been developed [6,8]. Many countries have introduced some of these vaccines into their immunization programs, which resulted in a marked reduction in the incidence of invasive diseases caused by vaccine-related serotypes/serogroups (e.g., >90% for Hib) [3,4,5]. Moreover, a significant change in serotypes/serogroups of meningococcus, pneumococcus, and *H. influenzae* causing invasive diseases, a phenomenon referred to as “serotype replacement”, has been observed after the use of conjugate vaccines in many populations [9,10,11]. 

In Serbia, the Hib vaccine was implemented in 2006 with vaccine coverage that exceeded 95%. For meningococci, no vaccine is routinely applied, however, since 2016 a quadrivalent conjugated vaccine for the prevention of A, C, Y, and W-135 serogroup-related infections has been recommended for use in high-risk individuals only (≥2 years old), but the recommendation has recently been changed to include children 9 through 23 months of age. PCVs have been available since 2009, but the coverage rate was low until April 2018, when PCV10 was introduced into the National Child Immunization Program. Regarding the surveillance of invasive diseases in Serbia, despite invasive meningococcal, pneumococcal, and *H. influenzae* related diseases being listed among the notifiable diseases, the existing epidemiological data are vague, due to under-reporting and overlapping diagnoses. According to the data collected by the National Institute of Public Health of Serbia, the reported incidence of bacterial meningitis in the past 5 years varied between 1.5 and 2.3/100,000 with missing data on the causative agents (http://www.batut.org.rs/index.php?category_id=140, accessed on 28 April 2021). Information on serotype/serogroup distribution for these pathogens is also scarce, and no comprehensive study on a broader scale has been done so far. For meningococci, the predominance of serogroup B has been reported [12], while existing data for pneumococci imply that circulating serotypes in some regions are similar to those observed in other countries before the introduction of PCVs and that antibiotic resistance might be significant in some serotypes [13,14,15,16]. However, none of the studies performed thus far addressed the distribution of serogroup/serotypes and antimicrobial resistance in invasive meningococcal and pneumococcal isolates at the country level, while information regarding *H. influenzae* is completely lacking.

Therefore, the aim of this study was to assess the serogroup/serotype epidemiology of invasive diseases caused by meningococcus and pneumococcus prior to the introduction of conjugate vaccines and to evaluate the impact of the Hib vaccine on serotype distribution of *H. influenzae* in Serbia. To this end, laboratory-based surveillance data from the National Reference Laboratory (NRL) for Meningococcus and Haemophilus and NRL for Streptococci were used to present serogroup/serotype distribution, antimicrobial resistance, and characteristics of invasive meningococcal, pneumococcal, and *H. influenzae* isolates from a child population in Serbia. The obtained results might provide valuable information for the evaluation of the present vaccination programs and the basis for the implementation of new vaccines in the future in this region.

## 2. Materials and Methods

Strains of *N. meningitidis*, *S. pneumoniae,* and *H. influenzae* isolated from primary sterile sites from children under 18, collected in the period from January 2009 to January 2018, were included in the study. Participating laboratories (*n* = 18) from different regions of Serbia performed the isolation and identification of invasive isolates using standard bacteriological procedures, and sent isolates to NRL for Meningococcus and Haemophilus and NRL for Streptococci. NRLs were responsible for identification confirmation, serotyping, and other typing procedures, as well as the antimicrobial susceptibility testing.

Identification confirmation of meningococcal isolates was done by Gram stain, positive oxidase, catalase and superoxol test, and BD BBL Crystal Neisseria/Haemophilus ID kit (Becton, Dickinson and Company, Sparks, MD, USA). Serogroups of meningococci were determined by slide agglutination using Pastorex antisera (Bio-Rad, Marnes-la-Coquette, France). Isolates that gave positive agglutination with B and C antisera were confirmed by conventional PCR with siaD-B and siaD-C primers, following a previously described protocol [17]. Pneumococci were reidentified based on colony morphology, Gram stain, optochin susceptibility, bile solubility test, and *lytA* gene confirmation by PCR [18]. Serotyping was performed with the Neufeld Quellung reaction using commercial antisera (Statens Serum Institute, Copenhagen, Denmark). *H. influenzae* isolates were reidentified using a BD BBL Crystal Neisseria/Haemophilus ID kit (Becton Dickinson and Company, Sparks, MD, USA), and serotyping was performed by slide agglutination with antisera (Statens Serum Institute, Copenhagen, Denmark).

Antimicrobial susceptibility testing (AST) was done by MIC test strip (Liofilchem, Roseto degli Abruzzi, Italy) for meningococci and *H. influenzae* isolates, and by E test (bioMerieux, Durham, NC, USA) for pneumococci. The following antibiotics were tested: penicillin, ceftriaxone, cefotaxime, ciprofloxacin, rifampin, and sulfamethoxazole-trimethoprim for meningococci; ceftriaxone, meropenem, ciprofloxacin, rifampin, and sulfamethoxazole-trimethoprim for *H. influenzae*; penicillin, cefotaxime, and erythromycin for pneumococci. Pneumococcal susceptibility to norfloxacin, sulfamethoxazole-trimethoprim, tetracyclines, chloramphenicol, and vancomycin was done by the disk diffusion method. Interpretation followed the recommendations of The European Committee on Antimicrobial Susceptibility Testing—EUCAST, when available, otherwise, the Clinical and Laboratory Standards Institute—CLSI standard was used [19,20]. For quality control, *S. pneumoniae* ATCC 49619, *H. influenzae* ATCC 49766, and *Staphylococcus aureus* ATCC 29213 were used.

For molecular typing of meningococci upon DNA extraction using peqGOLD Tissue DNA Mini Kit (Peqlab Biotechnologie GmbH, Erlangen, Germany), fine-typing was performed on a subset of randomly selected meningococcal isolates (27/53). DNA sequencing of genes encoding variable regions (VR) of the *porA* gene (VR1 and VR2) and the region of *fetA* gene encoding principal VR was done according to the published procedures [21,22]. Fine types were deduced from the obtained nucleotide sequences of *porA* and *fetA* genes, using the *Neisseria* Multi Locus Sequence Typing database (https://pubmlst.org/neisseria/, accessed on 1 January 2021). For molecular analysis of pneumococcal isolates, multilocus sequence typing (MLST) was performed as previously described [23]. It was done for a subset of penicillin and macrolide non-susceptible pneumococcal strains (57/67), randomly chosen within PCV13 serotypes. Sequence types (STs) were obtained at the MLST database (http://pubmlst.org/spneumoniae/, accessed on 28 April 2021). Additional information on molecular identification and typing is provided in the Supplementary Table S1.

Results were analyzed by standard statistical tests. Categorical data were compared using the Chi-square test and Fischer exact test, when appropriate. A *p*-value ≤ 0.05 was considered statistically significant.

## 3. Results

### 3.1. Clinical and Demographic Data and Characterization of Bacterial Isolates

#### 3.1.1. Clinical Diagnosis, Gender, Age, and Origin of Isolates

During the study period, a total of 53 *N. meningitidis*, 138 *S. pneumoniae*, and 11 *H. influenzae* invasive isolates were obtained. The number of invasive isolates received by the NRLs per year varied from 2 to 10 isolates for meningococci and from 9 to 24 for pneumococci. Approximately a third of meningococcal isolates originated from each of three arbitral regions of Serbia (the capital city, Belgrade, Vojvodina province, and Central Serbia), while the majority of pneumococcal isolates were from Belgrade (~60%), and approximately 20% originated from each of the remaining two regions. Patient data on clinical diagnosis, gender, and age, together with the sample type for invasive isolates are presented in Table 1.

Meningococcal isolates were dominantly isolated from patients with meningitis, whereas the majority of pneumococcal and *H. influenzae* isolates were obtained from the blood of patients with bacteremia, sepsis, and bacteremic pneumococcal pneumonia. More than half of all invasive isolates were from male patients. Regarding patient age distribution, the majority of meningococci (58%), pneumococci (63%), and *H. influenzae* isolates originated from children aged under two, corresponding to the natural peak for the occurrence of these infections. Almost 40% of meningococcal isolates were from children under one. Among pneumococci, 66% and 38% of isolates from patients with sepsis (including occult bacteremia) and patients with meningitis, respectively, were obtained from children 6 months (m) to 2 years (y) of age. 

#### 3.1.2. Serogroup/Serotype Distribution of Isolates

**Figure 1:**
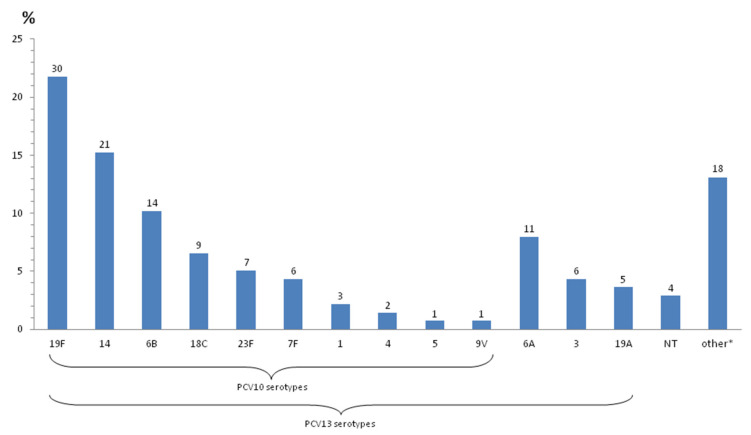
Serotype distribution of invasive pneumococcal isolates. Serotype distributions of invasive pneumococcal isolates are presented by a declining frequency of PCV10 serotypes and PCV13 serotypes (numbers of isolates for each serotype are presented above the bars). Other serotypes (*) refer to serotypes 23A (N = 3), 33F (N = 3), 9A (N = 2), 10A (N = 2), 9N (N = 1), 11A (N = 1), 12F (N = 1), 15B (N = 1), 15C (N = 1), 15F (N = 1), 17F (N = 1), and 22F (N = 1).

Within meningococcal isolates, serogroup B dominated, since as many as 44 isolates out of 53 belonged to this group (83%). Six isolates were of serogroup C (11.3%), while serogroups W135 and Y were only found in two (3.8%) and one isolate (1.9%), respectively. Among 138 pneumococcal isolates, 25 different serotypes were found (Figure 1). Eight most frequent serotypes (19F, 14, 6B, 6A, 18C, 23F, 3, and 7F), represented by at least six isolates each, encompassed more than 75% of all invasive strains. Apart from the youngest age group (0 to 6 m) with a rather small number of isolates, serotypes 19F and 14 dominated in all other age groups causing 37.9%, 41.3%, and 30.4% of IPD in children 6 m to 2 y, 2 to 5 y and 5 to 18 y of age, respectively. Three serotypes (14, 19F, and 18C) accounted for 43% of meningitis cases, while 47% of sepsis cases (including occult bacteremia) were related to serotypes 19F, 14, and 6B. Nevertheless, the association of any of the predominant serotypes with a particular clinical entity was not statistically significant. The coverage rate for PCV10 and PCV13 were 68% and 84%, respectively (Figure 1). Finally, among 11 invasive *H. influenzae* isolates there were six Hib strains, three non-type b strains, and two non-typeable strains (ntHi).

#### 3.1.3. Molecular Characterization of Isolates

Fine typing of meningococcal isolates revealed high antigenic diversity (Table 2). The most frequent antigenic fine type was P1.5-1,10-4 detected in seven serogroup B isolates. Among these strains, three shared the F5-8 type of FetA gene (data not shown). In order to further characterize isolated strains, MLST analysis was performed on pneumococcal isolates of vaccine-related serotypes. Twelve different STs were identified, and each of them was related to a particular serotype. The predominant ones were ST320 and ST271 (related to serotype 19F), ST15 (serotype 14), ST273 (serotype 6B), and ST81 (serotype 23F) (Table 2). Most of the identified STs were related to variants of the Pneumococcal Molecular Epidemiology Network (PMEN) international clones.

#### 3.1.4. Antimicrobial Resistance of Isolates

Results of antimicrobial susceptibility test showed that all *N. meningitidis* isolates were susceptible to ceftriaxone, cefotaxime, rifampin, and ciprofloxacin. However, more than half of the meningococcal strains (*n* = 29; 55%) showed moderate susceptibility to penicillin (Penms) with an MIC range of 0.094 to 0.25, whereas resistant isolates were not detected. The rate of penicillin non-susceptibility among serogroup B isolates was 52%. Sulfamethoxazole-trimethoprim non-susceptibility was also frequent: 77% (*n* = 41) and 11% (*n* = 6) of isolates were resistant and intermediate, respectively. Among serogroup B isolates, resistance was even higher, reaching 93%.

Among pneumococcal isolates, resistance (including both I—increased exposure and R—resistant categories) was common to beta lactams, erythromycin, clindamycin, sulfamethoxazole-trimethoprim, and tetracyclines (Table 3). In contrast, the incidence of norfloxacin and chloramphenicol resistance was low, and all strains were susceptible to vancomycin. The overall rate of penicillin non-susceptibility (as defined by MIC > 0.06 μg/mL) was 66.7% and it differed between meningitis and non-meningitis isolates (74% and 65.5%, respectively). A significant proportion of isolates showed cefotaxime non-susceptibility (37%). Erythromycin resistance was also common (55%) and a high proportion of isolates (48.5%) was both penicillin and macrolide non-susceptible. Of note, an alarmingly high proportion of isolates (28.2%) was resistant to at least five antibiotic classes (beta lactams and macrolides, and three of the following classes: lincosamides, tetracyclines, sulfamethoxazole-trimethoprim, chloramphenicol, and fluoroquinolones). The prevalence of antimicrobial resistance varied among isolates belonging to different serotypes (Table 3). The highest rates of penicillin non-susceptibility were found among 19F, 14, and 23F isolates, while cefotaxime non-susceptibility was widespread in serotypes 19F and 14. On the other hand, macrolide resistance was most common among isolates of 6A, 6B, and 23F serotypes. The rate of fluoroquinolone resistance was low, and it was detected among serotypes 14, 19F, 6A, and one NT strain. The frequency of penicillin and macrolide co-resistance was high among strains belonging to serotypes 6A (100%), 23F (100%), 6B (71%), 19F (70%), and 14 (48%). In addition, these five serotypes (19F, 14, 6A, 6B, and 23F) represented >90% of strains resistant to at least five antibiotic classes. Overall, PCV10 and PCV13 serotypes contributed a huge part to the total resistance rate of invasive isolates (Table 3). Among PCV10 serotypes, resistance was significantly more common to cefotaxime, clindamycin, sulphamethoxazole-trimethoprim, and tetracycline compared to the non-PCV10 serotypes (*p* < 0.01 for each antibiotic). Likewise, in PCV13 serotypes, significant association with non-susceptibility to penicillin, cefotaxime, erythromycin, clindamycin, sulphamethoxazole trimethoprim, and tetracycline was observed when compared to the non-PCV13 serotypes (*p* < 0.01, except for penicillin and clindamycin, *p* < 0.05).

Regarding *H. influenzae*, all isolates were susceptible to ceftriaxone, cefotaxime, rifampin, and ciprofloxacin, and resistance was detected only to sulfamethoxazole-trimethoprim in three strains.

## 4. Discussion

This study presents the first all-inclusive data from Serbia on invasive meningococcal, pneumococcal, and *H. influenzae* isolates from children gathered through the laboratory-based surveillance system. It highlights the serogroup/serotype distribution and antimicrobial resistance of two major invasive disease-associated pathogens, meningococcus and pneumococcus, prior to the introduction of conjugate vaccines that might be helpful for the monitoring of the impact of immunization on disease epidemiology over time.

Our data showed that among *N. meningitidis* strains causing meningitis and sepsis as major invasive meningococcal diseases (IMD) in Serbia, serogroup B predominates, as the majority of isolates (>80%) belonged to this group. In other European countries, serogroup B also prevails, followed by serogroup C, with fluctuations depending on a region, time period, and availability of vaccines [5,24]. Epidemiological survey of IMD in EU countries from 2004 to 2014 revealed that the majority of cases in all age groups were caused by serogroup B (74% in total), with decreasing trends of serogroup C-related IMDs in countries with meningococcal C conjugate (MCC) vaccine introduced into immunization programs [4]. Although the MCC vaccine has not been applied in Serbia, serogroup C was rather rare (11%) and its low incidence could be due to an overall small number of isolates, or might indicate the true distinctiveness of meningococcal epidemiology in this region. Indeed, similar serogroup distribution was recorded in the past in some countries in the region, such as Slovenia [25]. As for serogroup Y, despite its increasing trend in many parts of Europe, in Serbia, its incidence is rather low, as already documented in Southern Europe [26]. The W-135 serogroup was also rare in our study, although some countries reported much higher incidences of this serogroup [27]. Concerning antimicrobial susceptibility of our meningococcal isolates, moderate susceptibility to penicillin was detected at a higher frequency compared to other regions in Europe [28] and nearby countries [29]. Penms strains were found among all serogroups, particularly, a high-frequency was recorded among isolates of serogroup B. This observation is in contrast to data from other areas, where penicillin non-susceptibility is primarily related to C and other non-B serogroups [28]. Among 27 isolates that were fine typed in our study, we noticed the predominance of the P1.5-1,10-4 type, which was not among the most prevalent ones in Europe [30]. Three of those isolates shared the F5-8 type of the *fetA* gene implicating a possible clonal propagation. As for fine type P1.7-2.4, related to the multicomponent protein-based vaccine (4CMenB), it was not identified among our isolates, although one strain contained the PorA VR2 4 variant. A recently published study of IMD isolates from different European countries revealed a complete absence or low frequency of vaccine-related PorA VR1 7–2 and PorA VR2 4 isolates in our region, implying a sporadic occurrence of these types in this part of Europe [30]. Nevertheless, since porin PorA represents only one out of four different peptide components in the 4CMenB, the sequence analyses of genes encoding other peptides, namely factor H binding protein (fHbp), Neisserial heparin-binding antigen (NHBA), and Neisseria adhesin A (NadA), are needed to further analyze circulating meningococcal strains in our country and predict potential vaccine coverage. To this end, the Bexsero antigen sequence typing (BAST) scheme that enables characterization of vaccine antigen variants [31] or meningococcal antigen typing scheme (MATS) that identifies the expression of vaccine antigens on circulating strains could be used [32]. Estimates show that 4CMenB protection is around 80% in most European countries [32], so comparable coverage could be expected in Serbia, as well. Alternatively, the meningococcal antigen surface expression (MEASURE) assay that monitors fHbp surface expression and accessibility on intact bacteria by flow cytometry may be used to estimate the breadth of coverage of another recombinant meningococcal vaccine, bivalent rLP2086. Therefore, a new study with a higher number of isolates and the use of BAST/MATS or MEASURE analyses is needed to assess whether the introduction of a vaccine against serogroup B could help reduce the burden of IMDs in this country [33].

Regarding invasive pneumococcal isolates from children, our results revealed that vaccine-related serotypes 19F, 14, 6B, 6A, 18C, and 23F dominate, which is in line with the data from the northern region of Serbia [14]. Such findings are not surprising, considering very low vaccination rates in our country in the past period. Among our isolates, the coverage of conjugate vaccines was high, i.e., 68% for PCV10 and 84% for PCV13, with the difference mainly due to a relatively high proportion of the 6A serotype (8%). Serotype distribution of strains causing IPD in Serbia resembles those in other European countries before PCV implementation [34], including our neighboring countries [35,36,37]. However, certain differences could be observed, such as the predominance of serotype 19F over serotype 14 that dominated in most other parts of Europe in the pre-PCV era [34], or the low frequency of vaccine serotypes 1, 4, and 9V. The observed discrepancies could be due to a relatively small total number of studied isolates that may have influenced the relative frequency of each serotype, but they may also be explained by the rate of antibiotic resistance of the prevailing serotypes. It is well established that antibiotic pressure could be a driving force for the propagation of resistant clones of pneumococci [38]. Considering that Serbia is a country with a high consumption of antibiotics including macrolides [13,39], it is plausible to assume that this could have led to a relative predominance of serotype 19F exhibiting a higher rate of macrolide resistance compared to serotype 14 in our study (73% vs. 57%, respectively). On the other hand, overuse of antibiotics in Serbia could also be the reason for a rather sporadic occurrence of serotypes 1 and 4 that were generally susceptible to antibiotics in our study (data not shown). As for serotype replacement, the so-called “new” serotypes/serogroups (19A, 12, 22, 33, 15, etc.) shown to emerge after PCV implementation in many European countries [40] were also detected in the present study, but at a low frequency. In particular, the 19A serotype was less frequent compared to the other regions in the world, where its incidence has increased, predominantly as a post-PCV7 effect [40,41]. A higher incidence of the 19A serotype has also been observed in some countries after the introduction of PCV10 [42]. However, PCV10 is shown to induce an antibody response against serotypes 19A (and 6A) not included in the vaccine and thus confer some level of cross-protection between related serotypes 19A/19F and 6A/6B [7]. In line with this observation, several studies demonstrated a protective effect of PCV10 against serotype 19A IPD in vaccinated children, whereas, in some countries where PCV10 was used, no changes in serotypes 19A and 6A IPD rates were observed [7,43]. Therefore, it is not clear whether the recently introduced PCV10 vaccination in Serbia will lead to a rise in the frequency of non-vaccine types, including 19A and 6A, and it is on the future surveillance follow-up studies to assess this.

One of the striking findings of the present study was the high level of antimicrobial resistance among invasive pneumococci, especially for penicillin, macrolides, and third-generation cephalosporins (67%, 55%, and 37%, respectively). The last is particularly worrisome, considering the empiric therapy for invasive infections in children. The observed resistance rates of the abovementioned antibiotics in our invasive isolates are higher than the rates annually reported by the Central Asian and Eastern European Surveillance of Antimicrobial Resistance (CAESAR) network by the World Health Organization, but lower than those recently observed in a Survey of Antibiotic Resistance (SOAR) in four Eastern European countries [16,44]. The noted discrepancies could be due to differences in sample type and/or origin between these studies, since CAESAR predominantly included invasive isolates from adults, whereas the SOAR study was done mostly on non-invasive strains of pneumococci. In addition, levels of clindamycin, tetracycline, and sulfamethoxazole-trimethoprim resistance were also elevated, as well the proportion of strains exhibiting resistance to multiple antibiotic classes. However, a large part of overall resistance observed in our invasive strains was related to serotypes included in both PCV10 and PCV13, namely 19F, 14, 6B, and 23F, so we might expect a significant decrease in resistance in the near future due to a widescale implementation of PCV10, as demonstrated before in other settings [45]. MLST analysis of resistant IPD isolates indicates that their population structure might be clonal and that the majority of strains are closely associated with the internationally disseminated multi-resistant PMEN clones. Among the 19F isolates, ST320 and ST271 related to the Taiwan19F-14/ST236 clone, and ST179 a variant of the Portugal19F-21/ST177 clone, were found. ST320 is broadly distributed worldwide, and related to both 19F and 19A serotypes, with the 19A/ST320 clone arising after PCVs implementation in many countries [46,47], but still not detected in Serbia. The highly resistant 19A isolates in our study were ST878, associated with the Denmark14-32/ST230 clone, which has been shown to emerge in the post-PCV period in some nearby countries [48], so future dissemination of this ST is possible in our country, too. Interestingly, some of our 19F/ST320 isolates exhibited high-level resistance to third-generation cephalosporins, a finding that has already been described in the Far East [49], but to the best of our knowledge, not in Europe so far. Other IPD isolates in our study related to PMEN clones (or their variants) were 14/ST15 (related to England14-9/ST9 clone), 23F/ST81 (Spain23F-1), 6B/ST273 (Greece6B-22), and 9V/ST156 (Spain9V-3). Interestingly, 6A isolates were homogenous and all related to ST473, and this association has already been described [50].

Concerning *H. influenzae*, the sample size of only eleven isolates collected by NLR in our study prevents any firm conclusions from being made. However, in light of more than 10 years of immunization practice and vaccine coverage that exceeds 95% in most years, a small number of isolates of Hib confirms that children are adequately protected with the current Hib immunization program. The epidemiology of invasive *H. influenzae* has changed following the introduction of the Hib vaccine in many regions, where an increase in *H. influenzae* invasive diseases due to NTHi and serotype a has been observed [10]. For instance, in England and Wales, the gradual increase in HTHi infections was detected reaching as much as 85% of all infections caused by *H. influenzae*, whereas Hib caused only 2% of infections [51]. Therefore, a similar scenario could be anticipated in Serbia too. In line with this notion is our finding of three non-type b and two ntHi among our invasive isolates, but more data are needed to verify this. 

The presented study is the first one to comprehensively analyze data from Serbia on invasive meningococcal, pneumococcal, and *H. influenzae* isolates in the pediatric population, albeit it has several limitations, the major one being the small number of invasive isolates included. The cause for this is linked to the clinical practice in our country that includes the sampling of blood cultures from hospitalized children only, hence, a number of cases (especially patients with occult bacteriemia), might have been missed. Also, children frequently receive empiric antibiotic treatment that may lead to false-negative culture results. Molecular approaches are not available for the diagnosis of sepsis, meningitis, and other IMDs and IPDs in Serbia further decreasing the number of confirmed cases. Finally, the laboratory participation in the research was voluntary, which restricted the number of isolates sent to the reference laboratories. Consequently, our sample size did not allow sensitive comparison between geographical and demographic characteristics of isolates. On the other hand, our study incorporated strains collected from children of all ages and from different regions of Serbia over a longer time frame, which could take into account the temporal changes in the trends of serogroup/serotype distribution and/or the resistance patterns.

## 5. Conclusions

This study provides valuable information on characteristics, serogroup/serotype distribution, and antimicrobial resistance of three major pathogens causing invasive infections in the child population and could be useful for the evaluation of vaccination policy and the integration of new vaccines into the national immunization program in Serbia. A large proportion of IMDs is caused by serogroup B meningococcus which implies that the implementation of vaccines for the prevention of serogroup B-related IMD might be beneficial. Regarding pneumococcus, the majority of IPD serotypes are included in the available PCVs, indicating that the recent introduction of PCV10 will likely result in a reduction of morbidity and mortality due to IPDs, as well as antimicrobial resistance in the near future. As for H. influenzae, though the effectiveness of the Hib vaccination is evident, a substantial burden of non-b and non-typeable disease remains. 

## Figures and Tables

**Table 1:** Clinical diagnosis, gender, age, and source of isolation of invasive isolates.

	*Neisseria meningitidis*		*Streptococcus pneumoniae*		*Haemophilus influenzae*	
	N	%	N	%	N	%
Diagnosis						
Occult bacteriaemia	/	/	29	21.0	/	/
Sepsis	19	35.8	51	37.0	8	72.7
Meningitis	34	64.2	37	26.8	2	18.2
Other	0	0	21	15.2	1	9.1
Source of Isolation						
Blood	19	35.8	93	67.4	8	72.7
Cerebrospinal fluid	34	64.2	37	26.8	2	18.2
Other	0	0	8	5.8	1	9.1
Gender						
Male	35	66	74	53.6	8	72.7
Female	18	34	64	46.4	3	27.3
Age Group						
0–6 m (<1 y *)	20	37.7	6	4.3	0	0
>6–24 m (1–2 y *)	1	20.8	82	59.4	9	82
>2–5 y	7	13.2	27	19.6	2	18
>5–18 y	15	28.3	23	16.7	0	0

* relates to *N. meningitidis*; m—month, y—year.

**Table 2:** Molecular characterization of isolates of *N. meningitidis* and *S. pneumoniae*.

*Neisseria meningitidis*			*Streptococcus pneumoniae*		
Serogroup	*PorA* Genotype	N	Serotype	Sequence Type	N
B	P1.5-1,10-4	7	19F	ST320	11
	P1.7,16	2		ST271	7
	P1.5-1	2		ST179	3
	P1.5-1,18; P1.5,2	9 *	14	ST15	9
	P1.7; P1.7-1;		6B	ST273	8
	P1.7-20; P1.7-20,4			ST8144	2
	P1.18-1,3; P1.18-1,34			ST5240	1
	P1.19,15		6A	ST473	5
C	P1.5,2; P1.5,2-59; P1.7	5 *	23F	ST81	7
	P1.7-2,15; P1.18-7,10		7F	ST230	1
W-135	P1.18-1,3	1	19A	ST878	2
Y	P1.5-1,2-59	1	9V	ST156	1
Total		27	Total		57

* each fine type was represented by one isolate only.

**Table 3:** Serotype-specific antimicrobial susceptibility of *S. pneumoniae* isolates.

Serotype-Specific Antimicrobial Non-Susceptibility Rate (%)										
Serotypes	N	%	Pen	CXT	Ery	CL	SXT	Tet	CHL	Nor
19F	30	21.7	86.7	66.7	73.3	80	70	80	0	3.3
14	21	15.2	85.7	61.9	57.1	61.9	85.7	38.1	28.6	9.5
6B	14	10.1	78.6	71.4	85.7	71.4	92.9	85.7	57.1	0
6A	11	8	81.8	0	90.9	9.1	9.1	9.1	0	9.1
18C	9	6.5	22.2	0	22.2	0	33.3	11.1	0	0
23F	7	5.1	85.7	57.1	85.7	85.7	71.4	85.7	42.9	0
3	6	4.3	33.3	0	33.3	16.7	0	0	0	0
7F	6	4.3	50	0	16.7	16.7	0	16.7	0	0
19A	5	3.6	60	40	40	20	40	40	0	0
Other	29	21	41.4	6.9	20.7	3.4	27.6	10.3	10.3	3.4
Total	138	100	66.7	37	55	42.8	52.2	42.8	14.5	3.6
Overall rate (%) of resistance attributable to vaccine serotypes										
PCV 10 Serotypes	94	68.1	72.8	92.2	73.7	88.1	83.3	89.8	85	60
PCV 13 Serotypes	116	84.1	89.1	96.1	92.1	93.2	87.5	94.9	85	80

Antibiotic abbreviations: Pen (penicillin), CXT (cefotaxime), Ery (erytromycin), CL (clindamycin), SXT (sulfametoxazole-trimethoprim), Tet (tetracycline), CHL (chloramphenicol), Nor (norfloxacin), and Van (vancomycin).

## Data Availability

The data presented in this study are available on request from the corresponding author. The information regarding meningococcal isolates is deposited at the European Meningococcal Epidemiology in Real-Time (EMERT) database (http://emgm.eu/emert/ accessed on 1 January 2021).

## Supplementary Materials

The following are available online at https://www.mdpi.com/article/10.3390/diagnostics11061059/s1, Materials and methods for molecular identification and typing of meningococcal and pneumococcal isolates and Supplementary Table S1. Primers used in PCR amplification and DNA sequencing.
