# Supplementary material for: A Laboratory-Based Surveillance Study of Invasive Neisseria meningitidis, Streptococcus pneumoniae, and Haemophilus influenzae Diseases in a Serbian Pediatric Population—Implications for Vaccination
Source: Diagnostics (Basel). 2021 Jun 9;11(6):1059. doi: 10.3390/diagnostics11061059 (PMC8228891; doi:10.3390/diagnostics11061059)
Supplement: Supplementary file 1 [file diagnostics-11-01059-s001.zip › diagnostics-1232655-supplementary.pdf]

Materials and methods for molecular identification and typing of meningococcal and pneumococcal isolates

For molecular confirmation and identification selected bacterial isolates were subjected to DNA isolation using commercial kits, namely peqGOLD Tissue DNA Mini Kit (Peqlab Biotechnologie GmbH, Erlangen, Germany) for meningococcal isolates and QiaAmp DNA Mini Kit (Qiagen, Hilden, Germany) for pneumococcal isolates. For meningococci, initial molecular confirmation was performed by conventional PCR with siaD-B and siaD-C primers, which amplify a region of the gene (*siaD*) encoding the sialic acid capsule polymerase using PCR MasterMix S (Peqlab, Biotechnologie GmbH, Erlangen, Germany), whereas for pneumococci, primers for Autolysin-Encoding Gene (*lytA*) were used with Taq PCR Core Kit (Qiagen, Hilden, Germany). Further molecular characterization of meningococci was done by PCR amplification and DNA sequencing of variable regions (VR) of target genes: *porA* (VR1 and VR2) and *fetA*. For molecular characterization of pneumococcal isolates, multilocus sequence typing (MLST) was performed by using primers amplifying a set of housekeeping genes. A list of primers is given in Supplementary Table S1.

Upon successful amplification and purification with MinElute PCR Purification Kit (Qiagen, Hilden, Germany), according to the manufacturer's instruction, PCR products were subjected to direct sequencing by BigDyeR Terminator v3.1 (Applied Biosystems, Foster City, CA, United States).

Meningococcal fine types were deduced from the obtained nucleotide sequences of *porA* and *fetA* genes, using the *Neisseria* Multi Locus Sequence Typing database (https://pubmlst.org/neisseria/) accessed on 28 April 2021, whereas pneumococcal sequence types (STs) were obtained at the MLST database (http://pubmlst.org/spneumoniae/), accessed on 28 April 2021.

Supplementary Table S1. Primers used in PCR amplification and DNA sequencing

	Gene Target	Nucleotide Sequence of Primer
Meningococci identification	*siaD* C	5-GCA CAT TCA GGC GGG ATT AG-3
	siaD C	5-TCT CTT GTT GGG CTG TAT GGT GTA-3
	siaD B	5-CTC TCA CCC TCA ACC CAA TGT C-3
	siaD B	5-TGT CGG CGG AAT AGT AAT AAT GTT-3
Meningococci fine typing	*porA* (P14)	5-GGG TGT TTG CCC GAT GTT TTT AGG-3
	porA (P22)	5-TTA GAA TTT GTG GCG CAA ACC GAC-3
	fetA (S1)	5-CGG CGC AAG CGT ATT CGG-3
	fetA (S8)	5-CGCGCCCAATTCGTAACCGTG -3
	porA VR1(U86)	5-GCC CTC GTA TTG TCC GCA CTG-3
	porA VR1 (435)	5-TTG CTG TCC CAA GGA TCA ATG GC-3
	porA VR2 (435)	5-GCC ATT GAT CCT TGG GAC AGC AA-3
	porA VR2 (773)	5-GGC ATA GTT CCC GGC AAA ACC GCC AT-3
	fetA S12	5-TTCAACTTCGACAGCCGCCTT-3
	fetA S15	5-TTGCAGCGCGTCRTACAGGCG-3
Pnemunococci identification	*lytA*	5-GGA GTA GAA TAT GGA AAT TAA TGT-3
	lytA	5-GCT GCA TAG GTC TCA GCA TTC CAA-3
Pnemunococci MLST	*aroE*-up	5-GCC TTT GAG GCG ACA GC-3
	aroE-dn	5-TGC AGT TCA (G/A)AA ACA T(A/T)T TCT AA-3
	gdh-up	5-ATG GAC AAA CCA GC(G/A/T/C) AG(C/T) TT-3
	gdh-dn	5-GCT TGA GGT CCC AT(G/A) CT(G/A/T/C) CC-3
	gki-up	5-GGC ATT GGA ATG GGA TCA CC-3
	gki-dn	5-TCT CCC GCA GCT GAC AC-3
	recP-up	5-GCC AAC TCA GGT CAT CCA GG-3
	recP-dn	5-TGC AAC CGT AGC ATT GTA AC-3
	spi-up	5-TTA TTC CTC CTG ATT CTG TC-3
	spi-dn	5-GTG ATT GGC CAG AAG CGG AA-3
	xpt-up	5-TTA TTA GAA GAG CGC ATC CT-3
	xpt-dn	5-AGA TCT GCC TCC TTA AAT AC-3
	ddl-up	5-TGC (C/T)CA AGT TCC TTA TGT GG-3
	ddl-dn	5-CAC TGG GT(G/A) AAA CC(A/T) GGC AT-3
